# Usability, Acceptability, Feasibility, and Effectiveness of a Gamified Mobile Health Intervention (Triumf) for Pediatric Patients: Qualitative Study

**DOI:** 10.2196/13776

**Published:** 2019-09-30

**Authors:** Riin Tark, Mait Metelitsa, Kirsti Akkermann, Kadri Saks, Sirje Mikkel, Kadri Haljas

**Affiliations:** 1 Institute of Psychology University of Tartu Tartu Estonia; 2 Triumf Research OU Tartu Estonia; 3 Department of Oncology and Hematology Clinic of Pediatrics Tallinn Children's Hospital Tallinn Estonia; 4 Department of Hematology and Bone Marrow Transplantation Clinic of Hematology and Oncology Tartu University Hospital Tartu Estonia

**Keywords:** psychological stress, coping skills, psychological feedback, mobile app, mHealth, mental health, chronic illness, cancer, pediatrics

## Abstract

**Background:**

Mental disorders are notably prevalent in children with chronic illnesses, whereas a lack of access to psychological support might lead to potential mental health problems or disruptions in treatment. Digitally delivered psychological interventions have shown promising results as a supportive treatment measure for improving health outcomes during chronic illness.

**Objective:**

This study aimed to evaluate the usability, acceptability, and feasibility of providing psychological and treatment support in a clinical setting via a mobile game environment. In addition, the study aimed to evaluate the preliminary effectiveness of the mobile health game.

**Methods:**

Patients aged 7 to 14 years with less than a year from their diagnosis were eligible to participate in the study. In total, 15 patients were invited to participate by their doctor. A total of 9 patients (age range: 7-12 years; mean age 9.1 years) completed the 60-day-long study in which the Triumf mobile health game was delivered as a digital intervention. In an engaging game environment, patients were offered psychological and treatment support, cognitive challenges, and disease-specific information. The fully digital intervention was followed by a qualitative interview conducted by a trained psychologist. The results of the interview were analyzed in conjunction with patient specific in-game qualitative data. Ethical approval was obtained to conduct the study.

**Results:**

Patients positively perceived the game, resulting in high usability and acceptability evaluations. Participants unanimously described the game as easy to use and engaging in terms of gamified activities, while also providing beneficial and trustworthy information. Furthermore, the overall positive evaluation was emphasized by an observed tendency to carry on gaming post study culmination (67%, 10/15). Psychological support and mini games were the most often used components of the game, simultaneously the participants also highlighted the education module as one of the most preferred. On average, the patients sought and received psychological support or education on 66.6 occasions during the 60-day intervention. Participants spent the most time collecting items from the city environment (on average 15.6 days, SD 8.1), indicative of exploratory behavior, based on the quantitative in-game collected data. During the intervention period, we observed a statistically significant decrease in general health problems (*P*=.003) and saw a trend toward a decrease in depression and anxiety symptoms.

**Conclusions:**

This study demonstrated that a game environment could be a promising medium for delivering comprehensive supportive care to pediatric patients with cancer alongside standard treatment, with potential application across a variety of chronic conditions. Importantly, the results indicate that the study protocol was feasible with modifications to randomized controlled trials, and the game could be considered applicable in a clinical context. By giving an empirical evaluation of delivering psychological support via the game environment, our work stands to inform future mobile health interventions.

## Introduction

### Background

The prevalence of chronic illnesses in children is on the rise, with up to a quarter of children in the general population having at least 1 chronic illness [1], including pediatric cancer. The situation is further complicated as up to 60% of chronically ill children have at least 1 co-occurring mental disorder [2], compared with the 10% to 20% prevalence in the general population [3,4]. Indeed, it has been well established that chronic illnesses predispose children for higher risk of developing mental disorders such as anxiety, depression, and behavioral problems [2,5].

Psychological support, although generally seen as an integral part of comprehensive and effective care [6,7], has not yet been unified across hospitals [8], leading to large differences in access to psychological care across regions. Differences in the availability and quality of psychological support increase the likelihood of leaving psychological problems unattended, which in turn may have long-term negative health effects and interfere with the treatment process of the underlying chronic illness [9]. Furthermore, untreated psychological problems may affect treatment compliance in the pediatric care setting [10,11] and may carry on into adulthood [12]. This attests to the need for the further understanding of psychological problems co-occurring with chronic illnesses, both at the level of risk factors and disease development but also in the search for effective novel intervention strategies. Timely and accessible evidence-based psychological support in the pediatric care setting might be a crucial factor in achieving desirable treatment outcomes.

The factors leading to mental burden among chronically ill patients are largely universal across different conditions, encompassing, for example, changes in daily routines, stressful states related to treatment procedures, and psychological uncertainty [2,13]. Relatedly, psychological problems are comparable between general and chronically ill populations, involving mostly symptoms associated with anxiety, depression, and behavioral problems [2,5,14]. Hence, various traditional intervention strategies aimed at reducing mental burden (eg, psychoeducational programs, solution-focused brief therapy, cognitive behavioral therapy [CBT], and mindfulness-based interventions) have been used interchangeably between chronically ill patients and patients without chronic illness [4,11,15-18].

In addition to traditional interventions, digital tools have been shown to be a promising avenue in delivering psychological support to patients. Currently, there are only a few mobile health (mHealth) apps for children with cancer that are publicly available, including: Pain Squad for pain management [19]; Re-Mission2 for patient empowerment [20]; and Super K where kids can fight cancer cells according to the game description [21]. However, these solutions have not integrated psychological care for patients and are primarily used for pain monitoring or empowering.

Rathbone et al [22] reviewed mHealth apps that used CBT principles for several psychological conditions and concluded that although apps can be effective tools in a health setting, the need for further studies that evaluate the effectiveness of various mHealth solutions is imminent [23]. Similarly, another recent review brought out the mental health app efficacy evaluation as a concern, showing that 38% of app store descriptions included phrases related to claims of effectiveness, whereas only less than 3% provided scientific evidence for such declarations [24]. Furthermore, previous findings also highlight the need to improve user engagement [25]. As an example of user engagement, the gamified version of smartCAT solution was found to be more effective than the nongamified version in delivering brief CBT treatment [26], therefore suggesting that gamification could be effective in achieving desired mHealth platform effectiveness targets. If the solution is effective, it can facilitate desired behavior changes [27] and ultimately lead to improved health outcomes.

Against this background, this study aims to assess the usability, acceptability, and feasibility of a gamified mHealth intervention, Triumf, both as a whole and by its individual constituent components. Furthermore, this paper explores the preliminary effectiveness findings of the mHealth game, Triumf, whereas further analysis with a pretest-posttest design and without randomization of the participants is published as a master thesis [28].

### Mobile Health Game, Triumf

A newly developed digital health intervention, called Triumf, aims to reduce the negative psychological changes associated with chronic illness through an mHealth game. For clarity purposes, *intervention* or (mobile) *game* will also be used interchangeably to refer to the Triumf digital health intervention from hereon in. Importantly, the game has been designed in cooperation with pediatric patients with cancer, their parents, and care teams to determine illness-related burdening factors to develop a solution with maximum relevance to patients. Creating a game environment in collaboration with the key stakeholders has allowed building an intervention that approaches children in a way that is familiar to them. In addition, patients may be more empowered to comply with treatment if the intervention is delivered in a way that approaches the treatment process from a new angle [29]. By delivering care through a safe and familiar game environment, it is possible to fill in the gap between attractiveness and effectiveness, a common challenge in digital health solutions. Furthermore, as children in general are increasingly using their mobile devices for day-to day socializing and free-time activities, a new and still unused avenue for interacting, supporting, and educating pediatric care patients has been opened.

Together with the cooperative input from the partners and previous research literature, the intervention has been designed to serve as a basis to deliver effective psychological support that is universally applicable across different chronic illnesses, while maintaining the platform-level flexibility to consider disease- and region-specific differences. Thus, the overall gameplay, mechanics, and setup of the game and its principles are also applicable to other chronic illnesses, such as diabetes and asthma. The aim of the intervention was to offer patients psychological support and information about their health condition and emotions that would (1) help children to better understand their new health condition, (2) offer children external support to promote internal motivation to better cope with the illness, (3) offer children cognitive challenge and distraction, but also activity-based learning of healthy behaviors, (4) profile and screen the psychological well-being of children and offer psychoeducation and coping techniques accordingly, and thereby (5) support the formation of better self-understanding and constructive health behaviors, such as physical activity and diet. In short, through the continuous screening and support of various aspects, the intervention seeks to identify, prevent, and lessen the potential psychological problems and support behavioral change.

Triumf intervention covers various aspects of comprehensive care, and the game user experience is personalized and dynamic. The game learns and adapts with the user and offers individual and targeted experience. The intervention follows a predetermined structure in onboarding where the players are guided through the game narrative. Further gameplay, that is, accessing the educational module, entertainment games, and other elements of the intervention, is determined by the in-game choices made by the player. Furthermore, provision of psychological support is dynamically dependent on the individual emotional state of the patient.

#### Main Theoretical Background of the Game

An inferior understanding of emotions has been found to be a risk factor for developing psychopathology [30] and poor coping or adaptation to illness [13]. Furthermore, future health of the chronically ill individuals is related to general health behaviors. Thus, a shift toward healthier behaviors is crucial [31]. The interactive gamified setting in the Triumf intervention puts theories of emotions [32,33], coping [13], behavior, and behavior change [34] to practical use and presents as an educational module of the game. The intervention also consists of several mini games that include games related to the application of the in-game learned information and cognitive challenges, as well as entertainment games that offer cognitive distraction. The emphasis of the game is on the storyline—saving the Triumfland City by finding one’s inner superpowers and taming the Disease Monster—to achieve player engagement and connectedness, also bringing personal meaning to the game [35].

**Figure 1:**
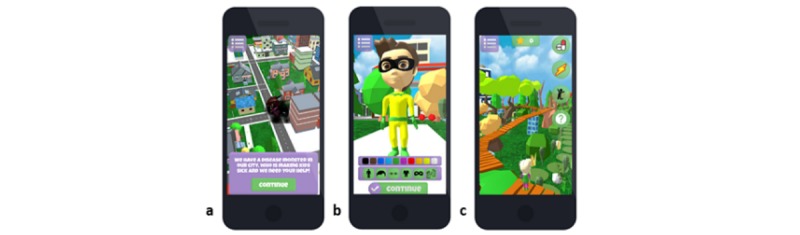
Visual representation of the intervention, displaying a screenshot: (a) from the introduction to the game (storyline), (b) from customization, and (c) from the obstacle course mini-game.

In addition, the game combines the Self-Determination Theory (SDT) and the Player Experience of Need Satisfaction model [36] to increase autonomy and the player’s competency experience in the game. SDT suggests that when the 3 core needs, autonomy, competence, and relatedness, are satisfied, they promote psychological health and intrinsic motivation [37]. It is argued that enhancing SDT needs in a game results in motivation to play [36]. As described by Tark [28], the Triumf intervention could enhance the following: (1) autonomy through offering noncontrolling guidelines and flexibility in choosing the flow of tasks and goals and by using in-game rewards as feedback instead of behavior-controlling mechanisms [36]; (2) competence through broadening knowledge about the illness and the importance of treatment adherence by using rewards and praise for successfully completed health-related actions and by keeping the players optimally challenged (eg, the possibility to choose the difficulty level in mini games) [36]; and (3) relatedness by creating an environment the player can relate to (eg, inclusion of illness-related but also regular child activities) and play against the game (eg, interacting with and helping city kids and playing tic-tac-toe against artificial intelligence). The visual representation of the 3D game, Triumf, is presented in Figure 1 and Multimedia Appendix 1, and individual modules and their theoretical background are presented in Table 1.

**Table 1:** Overview of the intervention modules.

Module		Rationale	Description
**Screening module**			
	Mental state	Profiles and screens to create preconditions for support [38]	Questions to form the player’s profile are prompted during the onboarding of the game. After the onboarding, the question how one feels at the moment is prompted daily. Two or more questions per day about symptoms of depression, anxiety, attention problems, and general health (well-being) are prompted depending on the player’s profile. In addition, well-being questions are accessible to the players throughout the game, ie, more than 2 questions per day can be answered by the player.
	Motivation and attitudes	Offers more specific psychological targets [36,39]	SDT^a^ questions about general attitude toward health, autonomy, competence, and relatedness are monitored once a week.
**Educational module**			
		Offers relevant information about the illness, treatment rationale, potential side effects, and hospital environment so that the child could be motivated and an informed participant in his or her treatment process [39,40]	After the onboarding, educational module is accessible to the player throughout the game. The topics are not presented in a predetermined order, ie, they appear based on the choices made by the player. The educational module presents each topic at 2 levels. First, the general introductory overview of the topic at hand is presented to the player. After the completion of the introductory level, more challenging in-depth educational description about the topic is presented to the player. This is followed by self-control questions that allow for the assessment and feedback about the acquisition of new information.
**Support module**			
	Psychological support	On the basis of the child's profile, the module offers psychoeducation and coping techniques [4,11,15-18,41]	Symptoms of depression, anxiety, attention problems, and general health questions all have 3 possible answers—sometimes, often, and rarely—based on which psychoeducation, psychological techniques, or praise is offered.
	Health behavior change	Motivates children to learn and engage in health-promoting behaviors through an educational module and content-relevant mini games (eg, a mini game that reminds the child to keep oneself well hydrated) [27,34]	Progressing in certain mini games requires applying information learned in educational module, which supports the motivation to engage in healthy behaviors and facilitates the consolidation of the acquired information.
	Emotion regulation	Helps children learn about identifying and regulating emotions [32,33,42,43]	Educational module includes information about 6 basic emotions, which the player also has to recognize in city kids. The circumplex model helps player to become better at identifying and relating to various emotional states. To be more specific, circumplex model, based affective state-space, gives the player a mental model to understand and relate to different specific emotional states, thus possibly facilitating cognitive top-town emotional regulation.
	Coping	Helps children better understand their new health situation [13]	Educational module information about one’s health situation and treatment procedures helps to normalize the daily challenges, wherein the support module offers ways how to cope with those challenges.
**Mini games module**			
	Storyline	Engages and fosters learning [35]	The storyline is introduced to the player during the onboarding.
	Activities common among children	Offers regular activities experience through entertaining games (eg, football), as children may be excluded from their social environment	Mini games are always accessible to the player after the onboarding.
	Cognitive distraction and cognitive challenge	Offers distraction and challenges through mini games such as puzzles, tic-tac-toe, and memory game	Mini games are always accessible to the player after the onboarding.

^a^SDT: Self-Determination Theory.

#### Screening, Support, and Educational Module

The rationale of psychological support provided in the game is based on continuous screening and monitoring of the player (patient). Questions about symptoms of depression, anxiety, attention, and general health are included in the game, wherein the interval and number of questions is based on the player’s profile. Questions addressing symptoms of depression, anxiety, and attention problems are based on DSM-5 diagnostic criteria; general health items include questions about hygiene, physical health, healthy eating, and sleep. The profile is created during the onboarding and is based on the answers given to 8 questions about emotional and attention state [32]. The player, based on the profile, is then categorized in one of the following: in good psychological functioning (minimum amount of screening and psychological support), in need of psychological support for emotional problems (more frequent screening and psychological support), and in need of psychological support in more than one area (more frequent screening and psychological support). All support questions have 3 possible answers (sometimes, often, and rarely) and after each answer, psychoeducation, psychological coping techniques, or praise is offered. The content of the psychological support constitutes the gamified versions of established evidence-based therapeutic methods, including mindfulness as well as CBT-based techniques, relaxation methods, and breathing exercises—all of which are commonly and successfully used in the context of chronic illnesses [11,15,16,41].

**Figure 2:**
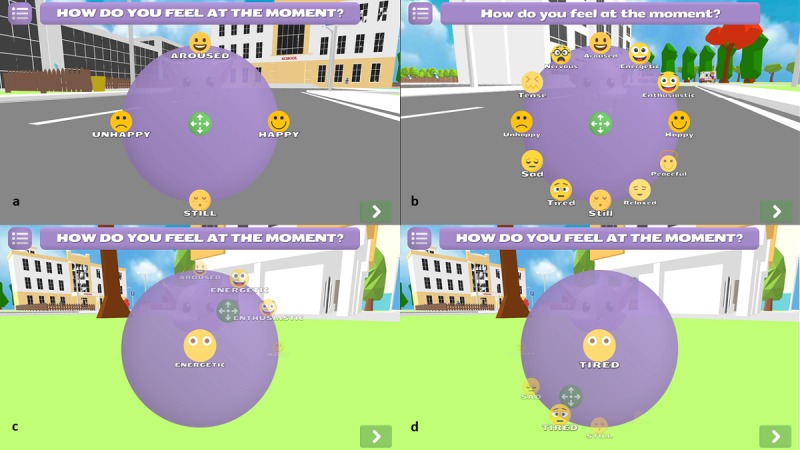
Presentation of specific emotional states in emotional state-space circumplex created by core affect dimensions valence and arousal: (a) displays appearance of the prompted question, (b) displays all possible answers, whereas (c) and d) display the appearance of answers when moving towards sectors of the circumplex.

In addition, questions about how one feels at the moment, how one slept the previous night, and one’s motivation and attitude toward health (SDT) are screened to complement the monitoring of the players’ well-being. A circumplex model [42] is used to probe the player’s emotional state by letting the player interactively indicate his or her emotional state by making a choice between specific emotional states that are situated in the affective state-space circumplex created by the interaction of core affect dimensions arousal (still-aroused) and valence (happy-unhappy). Specific emotional states have been indicated by the corresponding emoji figures (Figure 2). A question about how one slept the previous night compared with the average is prompted daily, as problems with sleep are common among chronically ill patients with accompanying psychological problems [33]. SDT questions about the general attitude toward health, autonomy, competence, and relatedness (health care climate; modified Treatment Self-Regulation Questionnaire [34]) are asked once a week to learn more about the needs and internalization of in-game learnt behaviors [39].

As one of the aims of the intervention is to induce behavioral change, it is essential to educate patients to support better coping, enhance resilience, and make informed decisions regarding their health and well-being [35]. By following the educational module, children learn about emotions, the illness itself, the treatment and its side effects, the care team, and social interactions with friends and family that may be affected by long-term hospital stay. The module is organized by levels that correspond to the Bloom taxonomy [40], which is often used as an underlying theoretical basis in pedagogics. Level 1 sets out to give information in a descriptive manner to facilitate initial learning. Level 2 gives further details on these topics. After reading the detailed material, several questions are asked to facilitate an understanding of the topic. Higher stages of the educational game include interactive modules to facilitate the carryover of new information from the semantic level to real-life related instrumental behaviors (ie, applying knowledge in content-relevant mini games, eg, identifying emotions of the citizens).

### Objectives of the Study

This study has 2 main objectives. First, to evaluate the usability, acceptability, and potential preliminary effectiveness of the intervention among pediatric cancer patients. Second, to assess the feasibility of the study protocol of administering the intervention without the randomization of patients.

Of note, the usability and acceptability assessments were collected both at the level of the whole game and at the level of constituent components. Furthermore, the preliminary assessment of possible beneficial effects on the well-being of pediatric patients with cancer was carried out.

To fulfill the main study aims, the following analytical steps were carried out: (1) assessment of the relations between participants’ game behavior, medical treatment, self-reported well-being, motivation and attitudes, sleep, and evaluations on the intervention, (2) assessment of the perceived usefulness, ease of use, and enjoyment of the intervention as a whole and at component level, (3) general evaluation of cooperation and process with hospitals, and (4) general evaluation of applicability of the game in clinical context.

## Methods

### Participants

**Figure 3:**
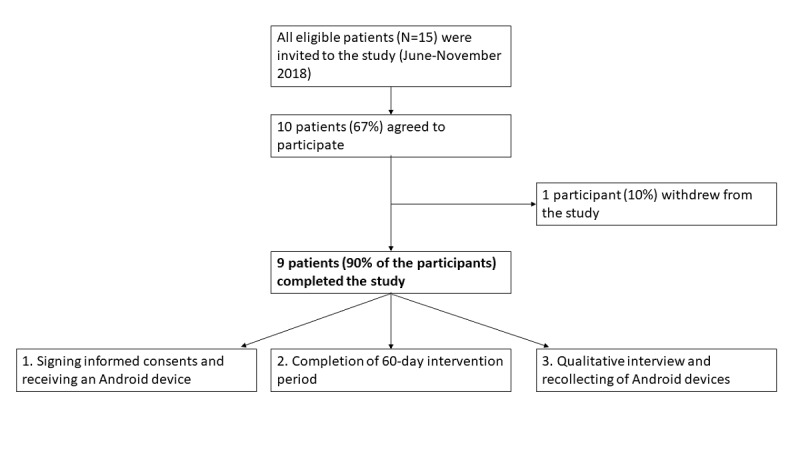
Participant flow diagram.

All pediatric patients with cancer aged between 7 and 14 years with a new or recurrent diagnosis of cancer, diagnosed no more than 1 year ago, were eligible to participate and were invited to the study (Figure 3). Participants were recruited by their medical doctors from Tallinn Children’s Hospital and Tartu University Hospital within a 6-month period starting from June 2018. Of the invited 15 children, 10 agreed (10/15, 67%) to participate in the study, with 1 participant withdrawing during the intervention owing to unfamiliarity with the game interface and without willingness to familiarize oneself. Thus, 90% of participants completed the study, and the sample used for analysis consisted of 9 pediatric patients with cancer with the average age of 9.1 years (SD 1.5; range 7-12), including 4 girls (44%) and 7 with Estonian as their native language (77%). Together with the withdrawn patient, 6 patients in total declined or withdrew their participation, with a mean age of 10.5 years (SD 2.4; range 7-14), 2 of them being girls (33%) and four (67%) having Estonian as their native language. Patients had various reasons for declining participation, where not being interested in the study or in mobile games was reported most frequently (4/6, 67%). However, the sample used for the final analysis did not statistically differ from the patients who decided not to participate or withdrew from the study (Table 2).

**Table 2:** Sample characteristics.

Variable		Patients who declined participation	Analytic sample	*P* value of declined patients vs analytic sample
**Sex, n (%)**				
	Girls	2 (33)	4 (44)	.67
	Boys	4 (67)	5 (56)	.67
Age (years), mean (SD)		10.5 (2.4)	9.1 (1.5)	.18
**Language, n (%)**				
	Estonian	4 (67)	7 (78)	.63
	Russian	2 (33)	2 (22)	.63
**Diagnosis category, n (%)**				
	CNS^a^ tumor	N/A^b^	1 (11)	N/A
	Leukemia	N/A	6 (67)	N/A
	Other	N/A	2 (22)	N/A
**Treatment status, n (%)**				
	Newly diagnosed	N/A	1 (11)	N/A
	On treatment	N/A	6 (67)	N/A
	Recurrent	N/A	2 (22)	N/A

^a^CNS: central nervous system.

^b^N/A: not applicable.

Each child and their parent gave written informed consent to participate in the study. The Research Ethics Committee of the University of Tartu (decision 283/T-32) approved the study protocol. Recruiting and participation in the study processes did not have deviations from the study protocol.

### Procedure

The study protocol involved a predetermined 60-day intervention period with a suggested 10 min of gameplay per day. The game was available in both Estonian and Russian. Android-operating smartphones were prepared and provided for the study period by the research team, and all participants had the option to continue the intervention following study completion using their own Android devices. Updates to the game were provided every other week to improve user engagement through new content. Technical support and information about game updates were offered to participants primarily via email.

Medical doctors recruited the patients to the study based on the eligibility criteria. Subsequently, information about the treatment and a health status for each participant were obtained from the medical doctors. This information included a general evaluation of the participant’s medical status during the study period, diagnosis and time from the diagnosis, notification of medical treatments that could affect mood, affect cognitive functions, or cause fatigue, time spent at the hospital according to the treatment plan, and outside planned treatment. Time from diagnosis, treatment that could affect participant’s capabilities, and total time spent at the hospital and at home were explored in the analysis. There were no deviations from the initial study protocol.

### Assessment

#### In-Game Assessment

Game activity and self-reported well-being, sleep quality, motivation, and attitudes were included in the analysis. The average scores for symptoms of depression, anxiety, attention problems, and general health across the study period were obtained as well-being indicators to observe possible relations with the SDT score (motivation and attitudes), game behavior, medical treatment, and intervention evaluation (derived from the qualitative interview). In addition, average scores in the beginning (first week average) and in the end (last week average) of the study period were compared to test the potential preliminary effectiveness of the intervention, with lower scores indicative of more problems.

Presentation of the sleep and SDT questions on a visual red-green gradient scale (slider) was coded on a 7-point scale such that higher scores indicate better than average sleep or better general attitude toward health, perceived autonomy, competence, and health care climate. A study period average and the first and last week average sleep score and total score of SDT (average of the questions answered) were calculated.

The progress in educational module was not included in the analysis owing to technical limitations despite being accessible to participants. The full play time from opening to closing of the intervention was also not observed owing to technological restrictions, and therefore, alternative variables indicating game activity were included in the analysis. These included total time spent in all mini games, total amount of collectables gathered (indication of exploratory behavior), total amount of interactions with citizens initiated (helping citizen maintain hydration and dental hygiene), and number of days when the game was used.

#### Evaluations on the Intervention and Study Process (Qualitative Interview)

A semistructured qualitative interview in the native language of the patient was held after completion of the 60-day study period. Trained psychologists conducted an oral qualitative interview with each participant and one of their parents. The interview was a structured evaluation of the usability and acceptability of the game following Marsac et al [44] and covered the following topics: (1) general attitude toward the game, including negative feedback, (2) participant’s subjective evaluation of the game (ie, whether it was beneficial, improved subjective well-being, helped to better follow the treatment plan, offered distraction from treatment, and was empowering), (3) evaluation of the intervention’s usability (whether it was easy to use, would they play more, were the guidelines clear, was there too much text, and did they learn something new), (4) acceptability of the game (whether it was fun to use, were the visuals nice, did the game have nice quests and mini games, nice characters, did it contain beneficial information, was the information accurate in their opinion, would they recommend the game to other children, and were the characters like oneself or like other children with cancer), and (5) evaluation on the study processes in general. There were no deviations from study protocol regarding the semistructured qualitative interviews. Questions regarding the general attitude toward and subjective evaluations of the game, as well as the evaluation of the study process, were asked in an undirected and neutral manner (eg, *What did you like about this game?*, *What you did not like?*, and *Do you think the game was beneficial?*). For usability and acceptability evaluations, participants were instructed to give yes/no answers to the neutrally read out statements (eg, *Triumf game was easy to use* and *Triumf game had too much text to read*). In addition, parents were asked to evaluate the general process of participating in the study. A summary of the interview answers, captured verbatim, was forwarded to the research personnel for analysis.

The aforementioned questions, to be included in the analysis, were coded as follows: clear *yes* answers as 1, expressions *yes, liked a little* or similar as 0.5, and clear *no* or *not really* as 0. Negatively formulated questions were reversed, which means higher scores indicated higher evaluations. General commentary on the game and evaluation of the study process were analyzed qualitatively.

From the subjective evaluations on the game, only 1 question (whether the game was beneficial) was answered by every participant (100%) and was rated beneficial unanimously. The remaining questions had missing values on several occasions, owing to some of the concepts (eg, *benefitting one’s well-being*) being difficult to understand by the young participants, and were thus excluded from further analysis. Usability questions were answered by all participants. From 8 acceptability questions, all participants answered only 2 questions, owing to difficulties in understanding by younger participants. One participant gave acceptability answers for half of the questions (ie, for 4) and was removed from further analysis concerning the acceptability aspect. To evaluate subjective evaluations, usability, and acceptability, the remaining data set was analyzed as described below.

### Statistical Analyses

International Business Machines SPSS version 20.0 [45] and R free software environment [46] were used for data analysis. Owing to this study’s exploratory nature, aiming to provide relevant research questions for further similar studies, we have reported tendencies as measure averages and used small group size–based statistical comparisons and correlations. Tendencies and statistically significant results based on our small sample provide valuable insights that could be meaningful and therefore could be more stringently tested in future studies that employ larger sample sizes.

In detail, a Pearson correlation was used to evaluate possible associations between age, total time of all mini games played, exploring around the city environment (total of collected items), helping city kids to maintain healthy behaviors (total of interactions), number of days when the intervention was used, treatment that could affect mood (in days), time spent at home (in days), time spent at the hospital (in days), time from diagnosis (in months), SDT average score, study period average depressive symptoms, study period average anxiety symptoms, study period average attention problems, study period average self-reported general health score, average sleep score, score of questions answered in the qualitative interview (questions answered yes divided by total number of questions answered), subscore of usability from the qualitative interview, and subscore of acceptability from the qualitative interview. Goodman and Kruskal *gamma* was used for exploring relations between sex and the aforementioned variables, as well as treatment complexity and noted variables. A paired sample (dependent) *t* test was used to evaluate group differences of continuous variables and a chi-squared test was used for evaluating differences in dichotomous variables. Covariates were not included in the analyses. A conventional cut-off point for 2-tailed significance (*P*<.05) was used.

## Results

### Sample Characteristics

Sample characteristics are presented in Table 2. The average time from diagnosis to recruiting was 5.3 months (SD 4.1, range 0-12 months). Participants received medical treatment that could potentially influence emotional state and cognitive functions on 12.7 days (SD 18.5, range 0-60), spent 17.2 days at the hospital (SD 15.8, range 0-37), and 42.8 days at home (SD 17.8, range 23-60). During the study period, 8 participants out of 9 (89%) received chemo or hormone therapy, and 1 participant received the last treatment right before the study period. Out of the 8 participants, 4 (44%) had a more complex treatment regimen (eg, presence of infection in addition to main diagnosis). The group of participants who had a more complex treatment versus the group who received regular treatment presented a statistically significant difference in total time spent playing mini games (*gamma*=−.70, *P*=.02), that is, participants with more complex treatment spent less time playing mini games. No systematic psychological support in the sample was received during the study period.

### In-Game Reported Well-Being Overview

Within the intervention environment, participants were divided into 3 categories on the basis of their profile of existing problems and strengths, resulting in 8 participants falling into the good psychological functioning category and 1 participant into the need for psychological support in more than 1 area category. On the basis of this, the majority of participants (8/9, 89%) received psychological screening and support approximately 2 times a day, with the exception of 1 patient, who received support more often. The participant who was categorized into the greater psychological support group had more depressive and anxiety symptoms, the lowest SDT score, and gave the lowest scores during the qualitative interview, but spent less time playing the game compared with the other group.

General comparisons (at the level of average scores) between the beginning and the end of the study period showed that there were no statistically significant changes in the in-game self-reported depression, anxiety, and attention problem symptoms, although a very slight improvement in depressive symptoms (from 1.15 to 1.19) and anxiety symptoms (from 1.46 to 1.50) was observed, accompanied with a very slight decrease in attention problems (from 1.31 to 1.29). For self-reported general health problems, there was a statistically significant change resulting in less problems (*t*_7_=−4.4; *P*=.003).

#### In-Game Reported Sleep

The average sleep score of the sample was 5.1 (SD 0.6) from a total of 7, indicating the best sleep quality compared with the average. Comparisons between the beginning (first week average) and the end (last week average) of the study period showed that sleep quality improved from the average score of 5.05 to 5.53, although the results were not statistically significant.

#### Motivation and Attitudes

Out of the 14 SDT questions, on average, 8.1 questions were answered (range 1-12) and the average SDT score for all participants was 5.4 (range 3.7-7). Comparisons between the beginning (first week average) and the end (last week average) of the study period showed that SDT scores remained stable over the course of the intervention ranging from 6.13 in the beginning of the study to 6.35 in the end. The lower average score of 5.4 for all participants was a result of greater variability across the study period.

#### Game Activity

On the basis of the quantitative in-game data, participants used the game on average on 20.2 days (SD 9.4), answered on average 66.6 (SD 51.6) support questions, and received support accordingly. Of specific modules, an obstacle course mini game was used on an average of 8.6 days (SD 4.3), a memory game on 6.1 days (SD 3.8), and a medication labyrinth mini game on 7.0 days (SD 3.9). Participants collected stars on 15.6 days (SD 8.1), water on 16.4 days (SD 9.2), and toothbrushes on 15.7 days (SD 8.8); and helped other citizens to stay hydrated and take care of dental health on 13.6 days (SD 7.6). The total average time spent playing mini games was 25.4 min (SD 19.6), total average amount of all collectables was 199.3 (SD 121.6), and helping citizens was initiated on an average of 74.2 times (SD 67.9).

Several statistically significant associations between the in-game data, medical treatment, and evaluations on intervention are presented in Multimedia Appendix 2. Symptoms of anxiety were positively correlated with usability evaluations (*r*=0.71; *P*=.03). General health problems and exploring around the city collecting different items was negatively correlated (*r*=−0.71; *P*=.03). In addition, less general health problems were reported when participants spent more time at the hospital (*r*=0.73; *P*=.03). Sleep score was positively correlated with acceptability evaluation of the intervention (*r*=0.67; *P*=.049).

There was a positive correlation between the SDT total score and time from diagnosing (*r*=0.70; *P*=.04). In addition, participant’s sex was negatively related to SDT score (*gamma*=−.80; *P*<.001), that is, boys had lower SDT scores.

Age and usability evaluation for the intervention were positively correlated (*r*=0.72; *P*=.03). Participant’s sex was positively related to the total amount of collectables (*gamma*=.60; *P*=.046), which means that boys collected more items. In addition, the more time participants spent at home during the study period, the more they explored around in the city collecting different items (*r*=0.87; *P=*.002). There were also statistically significant correlations between time from diagnosis and days spent at home (*r*=0.68; *P*=.04) and between game-related data (eg, the amount of collectables was related to helping citizens, *r*=0.71; *P*=.03).

### Evaluations on Intervention

Quantified usability and acceptability evaluations are presented in Table 3. All participants evaluated that the game was easy to use (100%) and 7 out of 9 (78%) would play it again. About 78% (7 out of 9) concluded that the instructions of the game were clear and that they learned something from the intervention, whereas 56% (5 out of 9) thought that there was too much to read in the educational module.

**Table 3:** Usability and acceptability evaluations for the intervention.

Evaluations		Value, n (%)
**Usability**		
	Number of participants in analysis	9 (100)
	Triumf game was easy to use	9 (100)
	Triumf game instructions were not confusing	7 (78)
	There was not too much to read in Triumf game	4 (44)
	I learned something new from Triumf game	7 (78)
	I would play Triumf game more	7 (78)
**Acceptability**		
	Number of participants in analysis	8 (100)
	I liked Triumf game visuals	7.5 (94)
	I liked Triumf game activities	8 (100)
	I liked Triumf game characters	7.5 (94)
	Triumf game contained beneficial information	8 (100)
	Triumf game information was trustworthy	8 (100)

Acceptability evaluation was based on the total of 8 participants’ answers. All of them (100%) concluded that they liked Triumf game activities and that the intervention contained beneficial information and that the intervention was trustworthy; 94% (7.5 out of 8) liked the visuals and the characters of the intervention.

The qualitatively analyzed subjective free form feedback included both positive and negative feedback. Interviews showed that each participant liked the different modules of the game the most. Specifically mentioned by the participants were the features of the memory game (1/9, 11%), obstacle course (2/9, 22%), medical labyrinth mini game (1/9, 11%), collecting stars (29, 22%), helping citizens (1/9, 11%), the well-being questions (1/9, 11%), educational module (3/9, 33%), and characters in general (5/9, 56%). The game in general was liked by 67% (6/9) of the participants. Interviews indicated that all the study participants used educational module. From negative aspects, different kind of preferences regarding the intervention were presented, for example, possibilities to access more buildings and use collectables in more advanced ways. The commonly reported critique indicated that the game was perceived to be too short for the 60-day intervention and that it was more interesting to play the game in the beginning of the study period until approximately half of the study period. Parents in general evaluated that the study process was smooth, and they were given sufficient amount of information.

When offered to continue playing the game following the study period, on their personal Android device, 6 of the participants out of 9 (67%) wished to do so. The reasons for the 3 participants not continuing were lack of a personal Android device (1 participant, 33%) and not interested (2 participants, 67%).

## Discussion

### Principal Findings

To the authors’ best knowledge, this was the first study to evaluate the usability, acceptability, potential preliminary effectiveness, and feasibility of a personalized digital health intervention that uses a game environment to deliver psychoeducation, coping techniques, and treatment support. This approach allowed the researchers to use gamified, personalized therapeutics to offer comprehensive supportive care to pediatric patients. Previous studies have repeatedly stressed that it is highly important to integrate pediatric oncology-psychology research and standard of care [47], as well as the comprehensive supportive care of other chronic illnesses [6]. This study gave support to the prospect of delivering supportive care through a digital mHealth game in addition to traditional methodology.

The main findings of the study showed that the patients positively perceived the game, specifically with regard to their engagement, liking of the intervention, and learnings from it. The quantitative data showed that the mini games and the support module were used the most, whereas the qualitative findings also indicated the use of the educational module by all patients. However, on the basis of the qualitative interview evaluations, all patients expressed their own opinions about the most favorite parts, showing that all participants found something valuable to them which, in turn, offered more personal content. In general, our findings are supported by previous literature which has found mHealth solutions to be well suited for children, most of whom are savvy technology users [23].

Out of the specific modules we found, on the basis of consistent monitoring during the intervention, the well-being of patients improved when considering general health, but did not change significantly for depression, anxiety, and attention problems. The evidence of no statistically significant change in mental health aspects could be explained by the generally stable mental state of the patients during the study period. Consistent monitoring is crucial for prevention and early detection of psychological problems as, for example, the intensity of the treatment and the change of health may influence the mental well-being. Furthermore, it could be expected that mental distress is experienced, even when the overall health status has improved, as noted in previous findings showing that psychological problems could last into adulthood [12]. The evidence that children receiving more complex treatment played fewer mini games requires further investigation of the potential different game behavior in this group of patients.

We also found that lower anxiety levels resulted in higher intervention evaluations, which could indicate that feeling less anxious during the intervention, and the qualitative interview may influence the child’s view and opinion about the intervention. Furthermore, longer stays at the hospital resulted in less general health problems, which could indicate that receiving treatment at the hospital, and therefore receiving greater monitoring by medical personnel and timely adjustment in diagnosis, could result in less-reported general health problems. Unexpectedly, better sleep was only related to higher evaluations on the intervention but not with mental or physical health status. However, the importance of sleep should still be emphasized, and sleep quality should still be continuously monitored to observe whether sleep disruptions indicate short-term or long-term problems [48,49].

General attitudes toward health showed that a higher SDT score was related to more time from diagnosis, indicating more positive attitudes and motivation toward health. However, boys had lower motivation and attitudes compared with girls and thus may need more support to reach desired health outcomes. Focusing on an SDT-based approach could be suggested, as previous research indicates that components of SDT are associated with improved self-care among chronically ill patients, and thus positively related to treatment compliance, quality of life, and other health-related outcomes [50]. In addition, using general theories such as SDT as guiding theories for Triumf game development efforts, many of which having been widely used to explain the facilitation of motivated health behavior in wide variety of previous studies [51], enables us to adapt the intervention for different chronic conditions. SDT theory is also associated with the concept of mental toughness that refers to an individual’s capacity to be consistently successful in coping with difficult life circumstances [52], one of the goals of Triumf intervention.

We also found that higher evaluations on the usability of the intervention were related to higher age, which could indicate that it was easier for older patients to understand the guidelines and text in the game. The youngest patients gave feedback that they did not understand some of the words used in the educational module. A revision of the text has been included in updates following the study, although already now the text is presented in levels, which allows younger patients to access more simple explanations compared with older ones who are able to access further details. Boys and those who spent more time at home engaged more in the exploratory use of the game. This could mean that patients feel more secure in the hospital setting and at home look for more support or that boys are more curious about the possibilities of the game.

The usability and acceptability results indicated high usability in general, and very high acceptability, with only too much reading being specifically brought out with regard to usability. In previous literature, a shortened text has been used [44], or alternatively, a more engaging way of presenting information could be implemented. The usability and acceptability findings are in accordance with previous literature that has highlighted that gamification is more engaging for users than intervention without gamification [26] and that using mHealth solutions is a valuable resource to deliver psychological techniques [22]. In general, the choice to collect feedback by constituent components of the game proved to be a valuable source of information, informing future studies about the presence of both strengths and *areas of improvement* at the same time.

The study protocol was generally feasible and was followed without deviations throughout the study period, although some amendments are necessary. Feedback from parents and medical doctors did not bring any modifications to the study protocol from the co-operation and communication perspective. As the questions of qualitative interview appeared difficult to understand for younger participants, a revision is needed. A simplification of the wording or more optimal amount of questions should be considered. In addition, transforming the oral interview format into a digital form could be more optimal for larger sample sizes. On the basis of the refusal and completion rates and necessary changes to the study protocol and the intervention itself, it could be concluded that the protocol is feasible for the randomized controlled trial, with minor modifications, and the game could be considered applicable in a clinical setting.

### Strengths

This study has several major strengths. First, the rationale behind the intervention has been clearly supported by previous research, which is central in understanding the included elements and therefore allows for the selection and design of gamified components that are potentially most effective [53]. Second, the intervention was built in a digital environment only, which indicates players’ engagement without external social encouragement and creates possibility to evaluate the cost-effectiveness of digital tools [53]. Moreover, a novel technological methodology to monitor and support patients in a timely manner was used. This interventional algorithm took individual responses and tailored the proposed support components accordingly. This study was conducted in 2 different hospitals, allowing to evaluate its effect independently of the hospital treatment context. Furthermore, it was observed that patients liked different modules of the game the most, suggesting that the personalized way of delivering psychological support is a preferred method, as we showed that all participants were able to use beneficial components of their liking. Taken together, our work informs future studies and contributes toward the development of effective mHealth interventions by giving the empirical evaluation of delivering psychological support in a health-focused digital game environment.

### Limitations and Further Directions

Several patients and their parents stated that the game was interesting for approximately half of the study period (30 days), followed by a decrease in engagement. A decrease in the intensity of use of mHealth solutions over time has been recognized [23], although this could be related to the intervention design, especially from the motivation and engagement perspective. On the basis of the above, the intervention period could be shortened in the randomized controlled study or the game could undergo significant updates throughout the 60-day intervention period to keep patients engaged with the intervention (a process that is already in place to an extensive degree). It is possible that a shorter intervention time may be sufficient to induce behavioral change as habit formation time is very individual [54]. For example, studies on improvement of physical activity using health apps have shown that shorter interventions are more effective, although findings on intervention effects over time are still scarce [55]. Taken together, a change to the study protocol includes the reconsideration of the length of the intervention.

As this study involved patients aged 7 to 12 years, future studies are needed to evaluate the game among other age groups (eg, 5-7 years and 12-16 years). The game was evaluated in the context of pediatric cancer; thus, the intervention could also be evaluated among children with other chronic illnesses.

In addition, it was found that the 1 individual who needed psychological support the most ended up using the intervention the least. There have been observations that more serious psychological problems may interfere with engagement with the game [26]. Considering that digital intervention studies have been conducted on patients with mild to moderate symptoms [53], it should be investigated further. To continue, amendments to the game should be made to accommodate the needs of those individuals who need psychological support the most. Thus, it was concluded that the intervention should be delivered in 2 steps. During the initial stage, only educational module and fun components of the game would be accessible. Subsequently, psychological intervention would follow. Through this 2-step approach, new information would be given in different stages of the intervention. This might result in higher compliance through reducing the initial load of information. The abovementioned modifications to the intervention would be implemented before the next study.

Although data on general health attitudes were collected, it was proposed that these findings would need reassessment, as reported general health problem results might have been dependent on whether prompted questions were about general health behaviors or related to the current health situation. Separate measures of the current health situation and general perceived health condition and behaviors should be considered.

### Conclusions

In conclusion, this study shows that delivering comprehensive supportive care through a game environment to pediatric patients is a feasible intervention strategy and is accepted by the patients and applicable in clinical context. This study showed that a game environment is a safe and engaging way of collecting real-time comprehensive data that can be used for personalized support.

## Appendix

Multimedia Appendix 1 Visual representation of the current Triumf mobile health game (version 1.0.5.2) displaying 4 screenshots of the mHealth intervention.

Multimedia Appendix 2 Correlations with confidence intervals.

## Abbreviations

CBT: cognitive behavioral therapy
mHealth: mobile health
SDT: Self-Determination Theory

## References

[1] van Cleave J, Gortmaker SL, Perrin JM (2010). Dynamics of obesity and chronic health conditions among children and youth. J Am Med Assoc.

[2] Butler A, van Lieshout RJ, Lipman EL, MacMillan HL, Gonzalez A, Gorter JW, Georgiades K, Speechley KN, Boyle MH, Ferro MA (2018). Mental disorder in children with physical conditions: a pilot study. BMJ Open.

[3] Kieling C, Baker-Henningham H, Belfer M, Conti G, Ertem I, Omigbodun O, Rohde LA, Srinath S, Ulkuer N, Rahman A (2011). Child and adolescent mental health worldwide: evidence for action. Lancet.

[4] Tark R, Jõeveer K, Tali A, Haljas K (2018). Tervisepsühholoogia: psühholoogi roll vähiravis. Eesti Arst.

[5] Hysing M, Elgen I, Gillberg C, Lie SA, Lundervold AJ (2007). Chronic physical illness and mental health in children. Results from a large-scale population study. J Child Psychol Psychiatry.

[6] American Diabetes Association (2018). Abridged for primary care providers. Clin Diabetes.

[7] Kirch R, Reaman G, Feudtner C, Wiener L, Schwartz LA, Sung L, Wolfe J (2016). Advancing a comprehensive cancer care agenda for children and their families: Institute of Medicine Workshop highlights and next steps. CA Cancer J Clin.

[8] Feudtner C, Womer J, Augustin R, Remke S, Wolfe J, Friebert S, Weissman D (2013). Pediatric palliative care programs in children's hospitals: a cross-sectional national survey. Pediatrics.

[9] Prince M, Patel V, Saxena S, Maj M, Maselko J, Phillips MR, Rahman A (2007). No health without mental health. Lancet.

[10] DiMatteo MR (2004). Variations in patients' adherence to medical recommendations: a quantitative review of 50 years of research. Med Care.

[11] Pai AL, McGrady M (2014). Systematic review and meta-analysis of psychological interventions to promote treatment adherence in children, adolescents, and young adults with chronic illness. J Pediatr Psychol.

[12] Secinti E, Thompson EJ, Richards M, Gaysina D (2017). Research review: childhood chronic physical illness and adult emotional health - a systematic review and meta-analysis. J Child Psychol Psychiatry.

[13] Compas BE, Jaser SS, Dunn MJ, Rodriguez EM (2012). Coping with chronic illness in childhood and adolescence. Annu Rev Clin Psychol.

[14] Merikangas KR, Nakamura EF, Kessler RC (2009). Epidemiology of mental disorders in children and adolescents. Dialogues Clin Neurosci.

[15] Beale IL (2006). Scholarly literature review: efficacy of psychological interventions for pediatric chronic illnesses. J Pediatr Psychol.

[16] Bennett S, Shafran R, Coughtrey A, Walker S, Heyman I (2015). Psychological interventions for mental health disorders in children with chronic physical illness: a systematic review. Arch Dis Child.

[17] Bond C, Woods K, Humphrey N, Symes W, Green L (2013). Practitioner review: the effectiveness of solution focused brief therapy with children and families: a systematic and critical evaluation of the literature from 1990-2010. J Child Psychol Psychiatry.

[18] Wiener L, Pao M, Kazak A, Kupst M, Patenaude A, Arceci R (2015). Pediatric Psycho-Oncology: A Quick Reference on the Psychosocial Dimensions of Cancer Symptom Management. Second Edition.

[19] (2014). App Store - Apple.

[20] (2015). Google Play.

[21] (2018). Google Play.

[22] Rathbone AL, Clarry L, Prescott J (2017). Assessing the efficacy of mobile health apps using the basic principles of cognitive behavioral therapy: systematic review. J Med Internet Res.

[23] Grist R, Porter J, Stallard P (2017). Mental health mobile apps for preadolescents and adolescents: a systematic review. J Med Internet Res.

[24] Larsen ME, Huckvale K, Nicholas J, Torous J, Birrell L, Li E, Reda B (2019). Using science to sell apps: evaluation of mental health app store quality claims. NPJ Digit Med.

[25] Ernsting C, Dombrowski SU, Oedekoven M, Sullivan JL, Kanzler M, Kuhlmey A, Gellert P (2017). Using smartphones and health apps to change and manage health behaviors: a population-based survey. J Med Internet Res.

[26] Pramana G, Parmanto B, Lomas J, Lindhiem O, Kendall PC, Silk J (2018). Using mobile health gamification to facilitate cognitive behavioral therapy skills practice in child anxiety treatment: open clinical trial. JMIR Serious Games.

[27] Fedele DA, Cushing CC, Fritz A, Amaro CM, Ortega A (2017). Mobile health interventions for improving health outcomes in youth: a meta-analysis. JAMA Pediatr.

[28] Tark R (2019). DSpace: University of Tartu.

[29] Fleming TM, Bavin L, Stasiak K, Hermansson-Webb E, Merry SN, Cheek C, Lucassen M, Lau HM, Pollmuller B, Hetrick S (2016). Serious games and gamification for mental health: current status and promising directions. Front Psychiatry.

[30] McLaughlin KA, Hatzenbuehler ML, Mennin DS, Nolen-Hoeksema S (2011). Emotion dysregulation and adolescent psychopathology: a prospective study. Behav Res Ther.

[31] Ford JS, Barnett M, Werk R (2014). Health behaviors of childhood cancer survivors. Children (Basel).

[32] Ekman P (2017). Facial expressions of emotion: new findings, new questions. Psychol Sci.

[33] Russell JA (2017). Mixed emotions viewed from the psychological constructionist perspective. Emotion Rev.

[34] Sheeran P, Klein WM, Rothman AJ (2017). Health behavior change: moving from observation to intervention. Annu Rev Psychol.

[35] Baranowski T, Buday R, Thompson DI, Baranowski J (2008). Playing for real: video games and stories for health-related behavior change. Am J Prev Med.

[36] Ryan RM, Rigby CS, Przybylski A (2006). The motivational pull of video games: a self-determination theory approach. Motiv Emot.

[37] Ryan RM, Deci EL (2000). Self-determination theory and the facilitation of intrinsic motivation, social development, and well-being. Am Psychol.

[38] Goodman R (2001). Psychometric properties of the strengths and difficulties questionnaire. J Am Acad Child Adolesc Psychiatry.

[39] Ryan RM, Patrick H, Deci EL, Williams GC (2008). Facilitating health behaviour change and its maintenance: interventions based on self-determination theory. Eur Psychol.

[40] Anderson LW, Anderson DR, Blooms BS (2001). A Taxonomy for Learning, Teaching, and Assessing: A Revision of Bloom's Taxonomy of Educational Objectives.

[41] Scholten L, Willemen AM, Grootenhuis MA, Maurice-Stam H, Schuengel C, Last BF (2011). A cognitive behavioral based group intervention for children with a chronic illness and their parents: a multicentre randomized controlled trial. BMC Pediatr.

[42] Yik M, Russell JA, Steiger JH (2011). A 12-point circumplex structure of core affect. Emotion.

[43] Suveg C, Zeman J (2004). Emotion regulation in children with anxiety disorders. J Clin Child Adolesc Psychol.

[44] Marsac ML, Winston FK, Hildenbrand AK, Kohser KL, March S, Kenardy J, Kassam-Adams N (2015). Systematic, theoretically-grounded development and feasibility testing of an innovative, preventive web-based game for children exposed to acute trauma. Clin Pract Pediatr Psychol.

[45] (2019). IBM - United States.

[46] (2019). The R Project for Statistical Computing.

[47] Armstrong FD, Reaman GH, Children's Oncology Group (2005). Psychological research in childhood cancer: the children's oncology group perspective. J Pediatr Psychol.

[48] Sivertsen B, Hysing M, Elgen I, Stormark KM, Lundervold AJ (2009). Chronicity of sleep problems in children with chronic illness: a longitudinal population-based study. Child Adolesc Psychiatry Ment Health.

[49] Dewald JF, Meijer AM, Oort FJ, Kerkhof GA, Bögels SM (2010). The influence of sleep quality, sleep duration and sleepiness on school performance in children and adolescents: a meta-analytic review. Sleep Med Rev.

[50] Williams GC, Patrick H, Niemiec CP, Williams LK, Divine G, Lafata JE, Heisler M, Tunceli K, Pladevall M (2009). Reducing the health risks of diabetes: how self-determination theory may help improve medication adherence and quality of life. Diabetes Educ.

[51] Deci EL, Ryan RM (2008). Self-determination theory: a macrotheory of human motivation, development, and health. Can Psychol.

[52] Bahmani DS, Hatzinger M, Gerber M, Lemola S, Clough PJ, Perren S, von Klitzing K, von Wyl A, Holsboer-Trachsler E, Brand S (2016). The origins of mental toughness - prosocial behavior and low internalizing and externalizing problems at age 5 predict higher mental toughness scores at age 14. Front Psychol.

[53] Hollis C, Falconer CJ, Martin JL, Whittington C, Stockton S, Glazebrook C, Davies EB (2017). Annual research review: digital health interventions for children and young people with mental health problems - a systematic and meta-review. J Child Psychol Psychiatry.

[54] Lally P, van Jaarsveld CH, Potts HW, Wardle J (2009). How are habits formed: modelling habit formation in the real world. Eur J Soc Psychol.

[55] Romeo A, Edney S, Plotnikoff R, Curtis R, Ryan J, Sanders I, Crozier A, Maher C (2019). Can smartphone apps increase physical activity? Systematic review and meta-analysis. J Med Internet Res.

