# Supplementary figures and images for: Usability, Acceptability, Feasibility, and Effectiveness of a Gamified Mobile Health Intervention (Triumf) for Pediatric Patients: Qualitative Study
Source: JMIR Serious Games. 2019 Sep 30;7(3):e13776. doi: 10.2196/13776 (PMC6792029; doi:10.2196/13776)

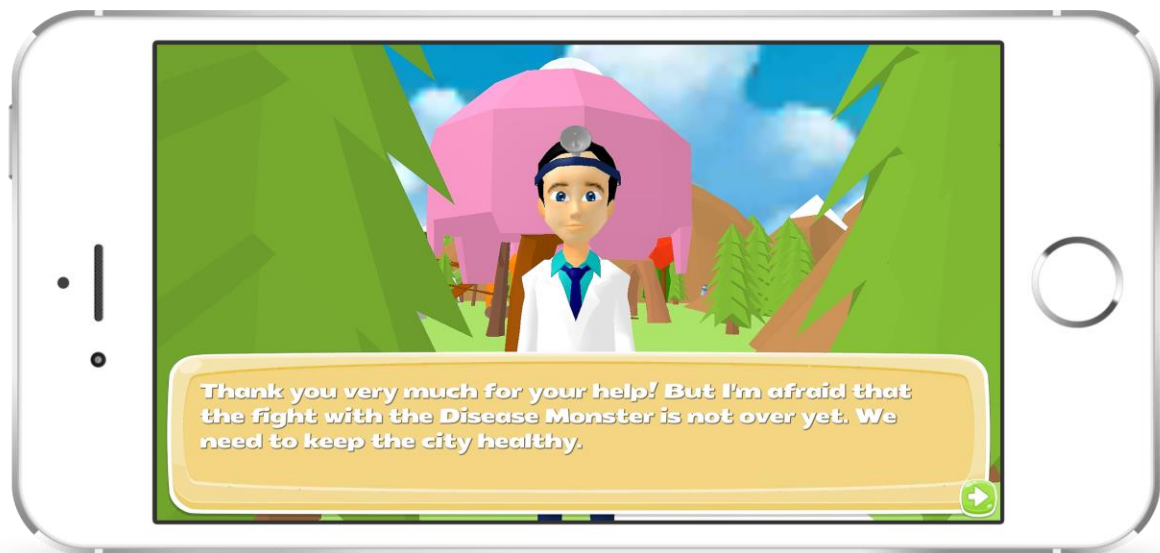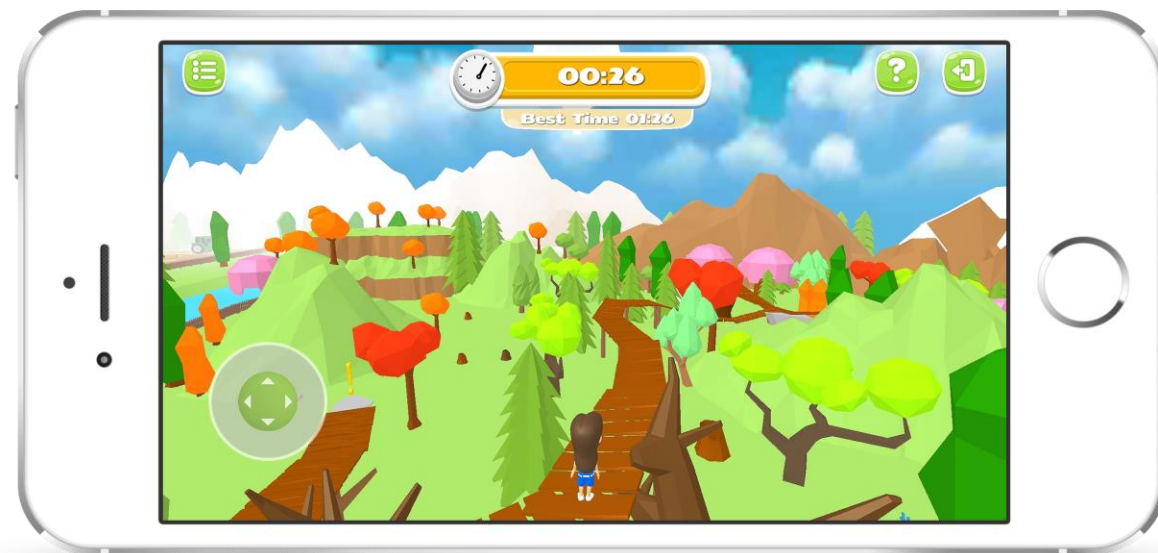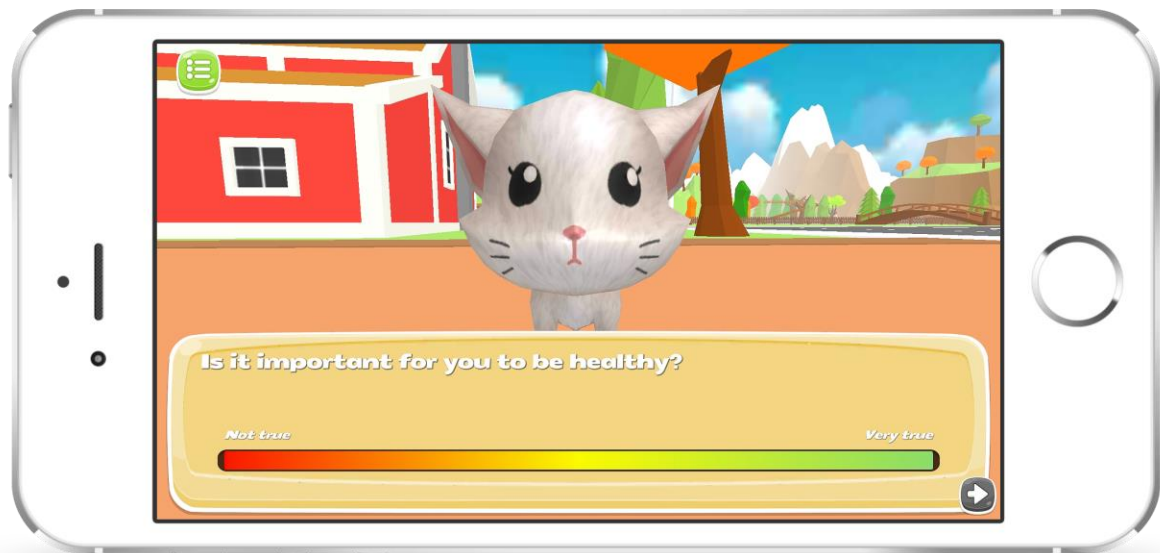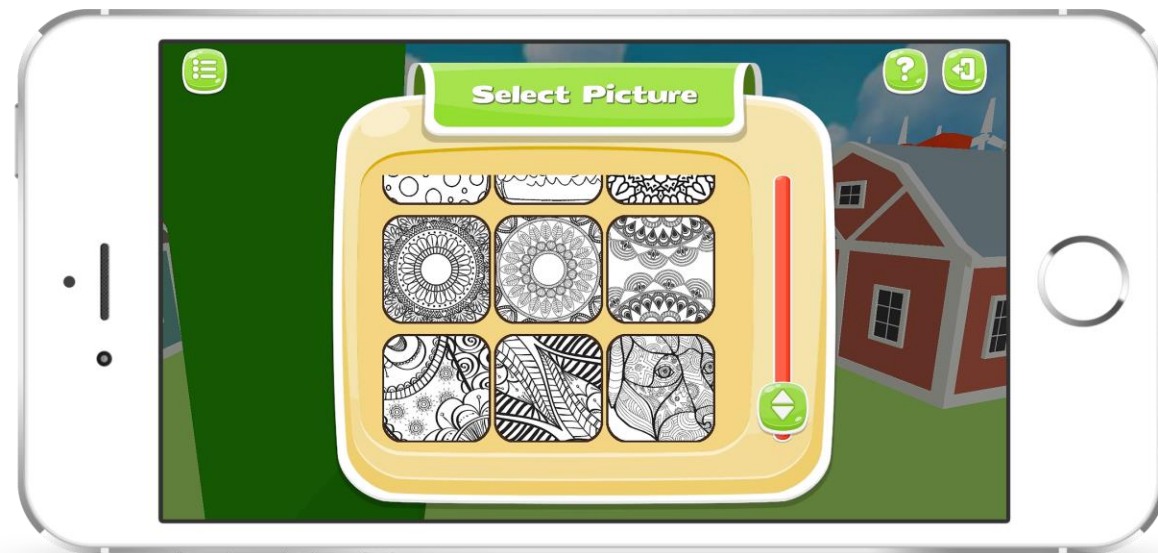

Supplement: Multimedia Appendix 1 [file games_v7i3e13776_app1.pdf]
