# Supplementary material for: Usability, Acceptability, Feasibility, and Effectiveness of a Gamified Mobile Health Intervention (Triumf) for Pediatric Patients: Qualitative Study
Source: JMIR Serious Games. 2019 Sep 30;7(3):e13776. doi: 10.2196/13776 (PMC6792029; doi:10.2196/13776)
Supplement: Multimedia Appendix 2 [file games_v7i3e13776_app2.pdf]

Multimedia Appendix 1. Correlations with confidence intervals

		game behaviour				treatment information				well-being						intervention evaluation		
	age	mini-games	collect-ables	helping others	game open	influencing mood	at home	at hospital	from diagnosis	SDT	dep-ressive	anxiety	attention	general health	sleep	overall	usability	accepta-bility
age	1.00	-0.64	0.00	0.04	-0.12	0.23	0.19	-0.19	-0.01	-0.01	0.27	0.35	0.34	-0.34	0.02	0.09	0.73	0.29
mini-games		1.00	0.41	0.21	0.43	-0.23	0.30	-0.30	0.50	0.23	-0.18	-0.13	-0.28	0.13	0.54	0.19	-0.53	0.37
collectables			1.00	**0.71***	0.40	-0.24	**0.87***	**-0.88***	0.41	-0.06	-0.09	0.26	0.36	**-0.71***	0.31	-0.17	0.16	0.07
helping others				1.00	**0.70***	-0.15	0.51	-0.51	-0.09	-0.30	0.31	0.62	0.40	-0.50	0.26	0.34	0.56	0.05
game opened					1.00	0.02	0.30	-0.30	-0.08	-0.34	-0.01	0.27	-0.02	-0.26	0.09	0.51	0.39	0.26
influencing mood						1.00	0.15	-0.15	0.36	0.28	0.21	-0.14	0.34	0.11	0.02	-0.16	0.13	0.20
at home							1.00	**-1.00***	**0.68***	0.29	0.04	0.07	0.35	**-0.73***	0.30	-0.22	0.16	0.33
at hospital								1.00	**-0.68***	-0.29	-0.04	-0.07	-0.35	**0.73***	-0.30	0.22	-0.16	-0.33
time from diagnosis									1.00	**0.70***	-0.02	-0.32	0.05	-0.18	0.51	-0.28	-0.40	0.55
SDT										1.00	0.49	-0.20	-0.08	0.18	0.48	0.09	-0.35	0.66
depressive											1.00	0.61	0.38	0.21	0.50	0.60	0.43	0.47
anxiety												1.00	**0.70***	0.06	0.51	0.54	**0.71***	0.18
attention													1.00	-0.08	0.35	-0.15	0.49	-0.08
general health														1.00	0.30	0.32	-0.36	0.11
sleep															1.00	0.40	-0.03	**0.70***
overall																1.00	0.41	0.59
usability																	1.00	0.13
acceptability																		1.00

* $p < .05$
